# Supplementary material for: Au-Embedded and Carbon-Doped Freestanding TiO2 Nanotube Arrays in Dye-Sensitized Solar Cells for Better Energy Conversion Efficiency
Source: Micromachines (Basel). 2019 Nov 22;10(12):805. doi: 10.3390/mi10120805 (PMC6953097; doi:10.3390/mi10120805)
Supplement: Supplementary file 1 [file micromachines-10-00805-s001.pdf]

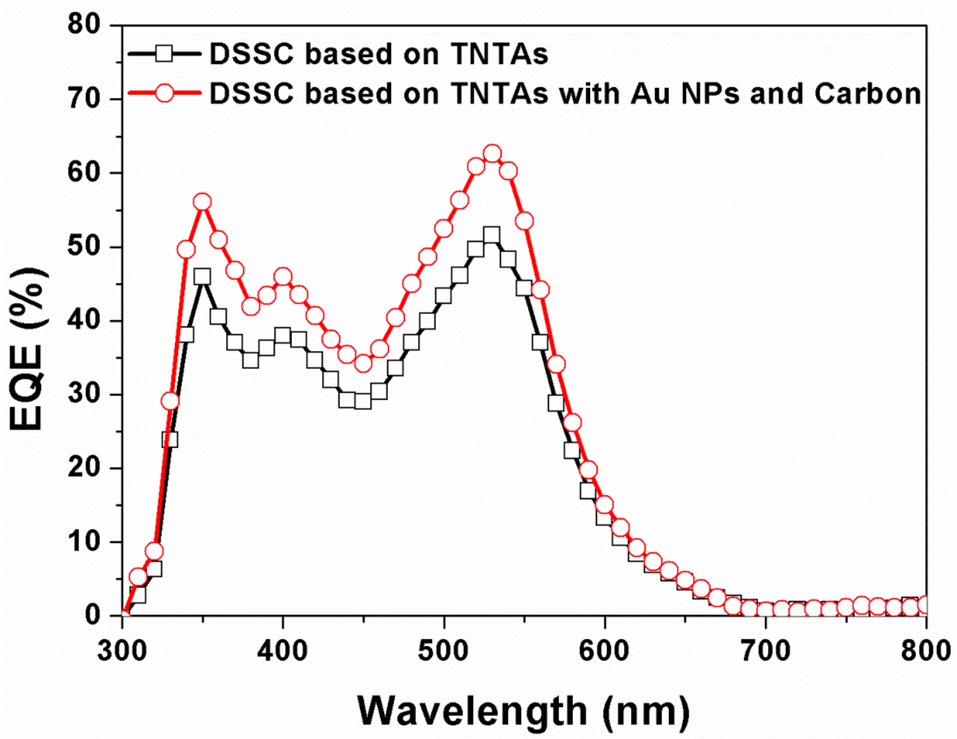

Figure S1. External quantum efficiency (EQE) of dye-sensitized solar cells (DSSCs). The black line is the DSSC based on the TNTAs and the red line is the DSSC based on the TNTAs with Au NP and carbon materials.

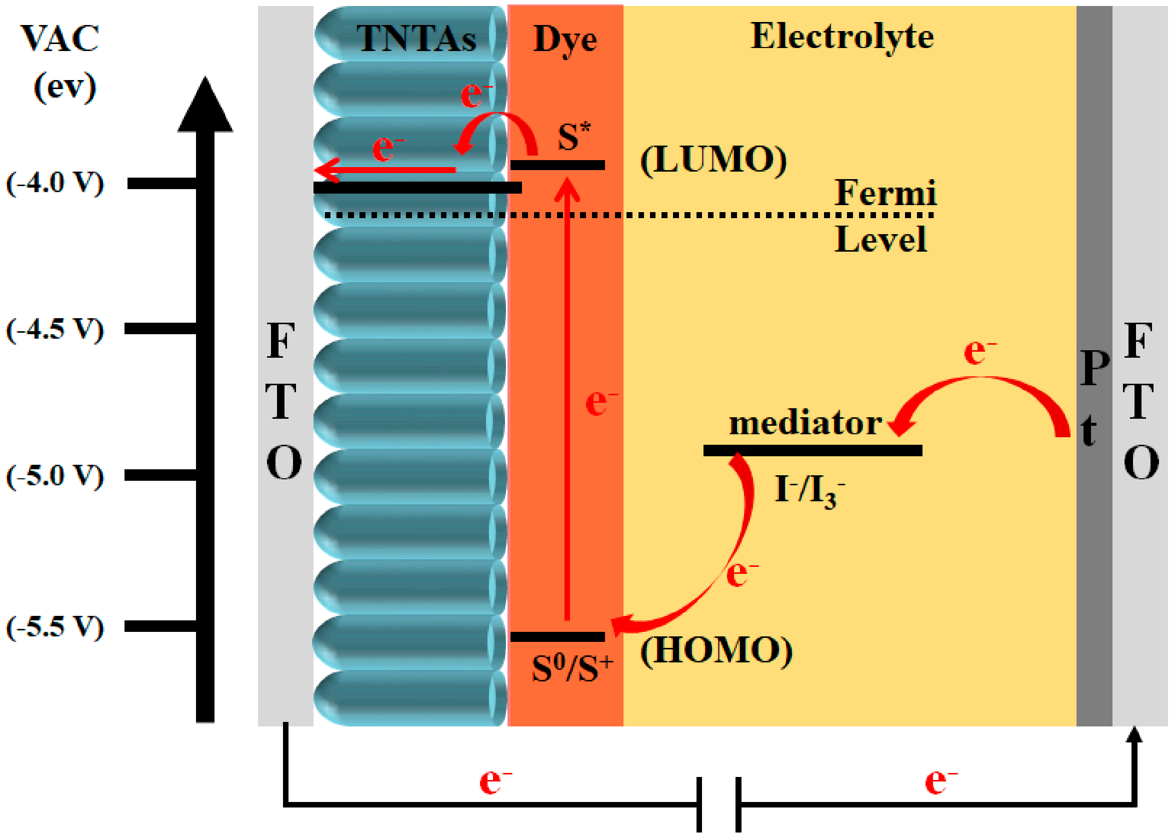

Figure S2. Energy band diagram of DSSC based on the TNTAs.
